# Depression, Suicidal Behaviour, and Mental Disorders in Older Aboriginal Australians

**DOI:** 10.3390/ijerph15030447

**Published:** 2018-03-04

**Authors:** Yu-Tang Shen, Kylie Radford, Gail Daylight, Robert Cumming, Tony G. A. Broe, Brian Draper

**Affiliations:** 1 St Vincent’s Hospital, Darlinghurst, Sydney, NSW 2010, Australia; 2 Neurosciences Research Australia, Randwick, Sydney, NSW 2010, Australia; cliffdaylight@gmail.com (G.D.); t.broe@neura.edu.au (T.G.A.B.); 3 Faculty of Medicine, University of New South Wales, Sydney, NSW 2010, Australia; 4 School of Public Health, University of Sydney, Sydney, NSW 2010, Australia; robert.cumming@sydney.edu.au

**Keywords:** late-life, depression, aboriginal, childhood, mental health, resilience

## Abstract

Aboriginal Australians experience higher levels of psychological distress, which may develop from the long-term sequelae of social determinants and adversities in early and mid-life. There is little evidence available on the impact of these on the mental health of older Aboriginal Australians. This study enrolled 336 Aboriginal Australian participants over 60 years from 5 major urban and regional areas in NSW, utilizing a structured interview on social determinants, and life-time history of physical and mental conditions; current psychosocial determinants and mental health. Univariate and multivariate analyses were utilized to examine the link between these determinants and current depressive scores and suicidality. There was a high rate of life-time depression (33.3%), current late-life depression (18.1%), and suicidal ideation (11.1%). Risk factors strongly associated with late-life depression included sleep disturbances, a history of suicidal behaviour, suicidal ideation in late-life and living in a regional location. This study supports certain historical and psychosocial factors predicting later depression in old age, and highlights areas to target for prevention strategies.

## 1. Introduction

Aboriginal Australians have been a marginalised population within Australian society. The impacts of early European settlement of Australia included disease, violence, and death upon the local Aboriginal Australian population, as well as the loss of traditional land through colonisation.

This protracted collective experience of trauma has resulted in a disruption of the social fabric of Aboriginal society [1]. The long-term sequelae have included psychosocial disadvantage, such as higher rates of unemployment, forensic history, childhood adversity, social inequity, poor education [2,3], early forced separation from parents, racism [4], diminished social and financial security, lack of community supports, and comorbid physical illness [5].

Significantly, psychological sequelae have included a higher lifetime prevalence of psychological distress, depression, and anxiety in the Aboriginal population compared to the general population [3], as well as higher rates of smoking and risky alcohol consumption [6]. There is also a higher rate of suicide and intentional self-harm [7].

### 1.1. Mental Health of Older Aboriginal Australians

There have been few studies examining the mental health of older Aboriginal Australians. In an urban sample of 1939 Aboriginal Australians aged 45 and over, Gubajhu et al. [3] found that there was a higher prevalence of psychological distress, lifetime depression, and lifetime anxiety than in the general population. The Aboriginal Australian sample was associated with having worse social and physical outcomes. There was no exploration linking the mental history or level of psychological distress with psychosocial or medical factors in this study.

In another sample of 250 Aboriginal Australians aged 45 and over living in the remote Kimberley region, Almeida et al. [8], utilised the KICA-dep depression screening instrument, which was correlated with a psychiatric interview; and demonstrated a point prevalence of depression 7.7%. This study also demonstrated a significant association between depression and cardiac problems (*p* = 0.012), with a trend towards significant association with female gender, diabetes, and kidney problems.

According to the ABS [9], there is a trend of increasing life expectancy in Aboriginal Australians over the years, from 61.9 years males, 66.1 years females in 1991–1996 estimates, to 69.1 years in males and 73.7 years in females in 2010–2012, with a projected increase of 0.2 year life expectancy per year in males, and 0.15 year per year in females. This will likely result in a rapidly ageing population beset by higher rates of physical and mental health comorbidities that need to be catered for in the near future.

### 1.2. Formative Experiences and Late-Life Depression

Recent research into the effects of childhood adversity has linked adverse experiences to later mental health outcomes. The Adverse Childhood Events (ACE) Study conducted in the US explored parental mental health, early home environment, and experiences of childhood abuse and found that emotional, physical, and sexual abuse have a strong relationship to later mental health outcomes, including depression and suicide in a dose-dependent relationship [10,11].

A recent study by Patten et al. [12] in two linked surveys in children and adolescents demonstrated a significant association between childhood adversity, including social cohesion, parenting influence, school environment, and stressful life events, with a higher rate of depression in young adulthood. An Australian study, the DEPS-GP study involving over 20,000 persons aged 60 years and over found that those who reported childhood sexual or physical abuse had an increased risk of poor late life mental health, with a cumulative effect of having both physical and sexual abuse having a higher risk [13]. Danese et al.’s [14] study also demonstrated a link between childhood adverse psychosocial factors with cumulative risk of nervous, immunological and endocrinological functioning. Recently, we reported an association between childhood adversity and late life depression, dementia, and other mental health outcomes in Aboriginal Australians [15].

There are other biological factors that lead to late life depression, including general physical health, vascular risk factors, such as coronary heart disease and cerebrovascular events, obesity, diabetes mellitus, and Parkinson disease, as well as cognitive disorders and other genetic factors. The concept of Allostatic Load [16,17] provides an explanatory framework to understand the contribution of long-term stress through the prolonged overactivation of the neuro-endocrine system coupled with sustained systemic stress imposed by the heightened humoral response on longer-term physical sequelae and emotional perturbations—which may lead to longer term vulnerability to mental illnesses such as depression. Contributing psychological factors encompass maladaptive personality traits, cognitive distortions, and learned helplessness. Social factors included life stressors, medical illness and functional disability, trauma, lower income and education, bereavement, lack of social support, and loneliness [18].

### 1.3. Aims of Current Study

The aims of the current study were to establish the prevalence of current depression, the lifetime prevalence of mental disorders in older Aboriginal Australians and to determine physical, psychological and social factors across the life span that were associated with depression in late life in this group.

## 2. Materials and Methods 

Participants were recruited from 5 different sites, in a culturally sensitive manner with the assistance of local Aboriginal research assistants across urban and regional areas in NSW using locally available lists and snowball recruitment. Two of the sites were from metropolitan Sydney (La Perouse, Campbelltown) and three sites were from the mid-north coast region (Kempsey, Nambucca, Coffs Harbour).

The inclusion criteria for participants were: aged 60 years and over, and identifying as Aboriginal and/or Torres Strait Islander Australian. The exclusion criteria included being incarcerated at the time of study, having had a recent stroke (within 3 months of assessment), and inability to obtain informed consent. Further detailed information on the recruitment process were reported in Radford et al. [19].

Ethics approval was obtained from the Aboriginal Health and Medical Research Council (AHMRC; 615/07), The University of New South Wales Human Research Ethics Committee (HREC 08003), and NSW Population & Health Services Research Ethics Committee (AU RED Ref: HREC/09/CIPHS/65; Cancer Institute NSW Ref: 2009/10/187). Written consent was obtained, or in cases of impaired capacity, assent from participant and consent from their guardian was obtained in their stead.

**Figure 1:**
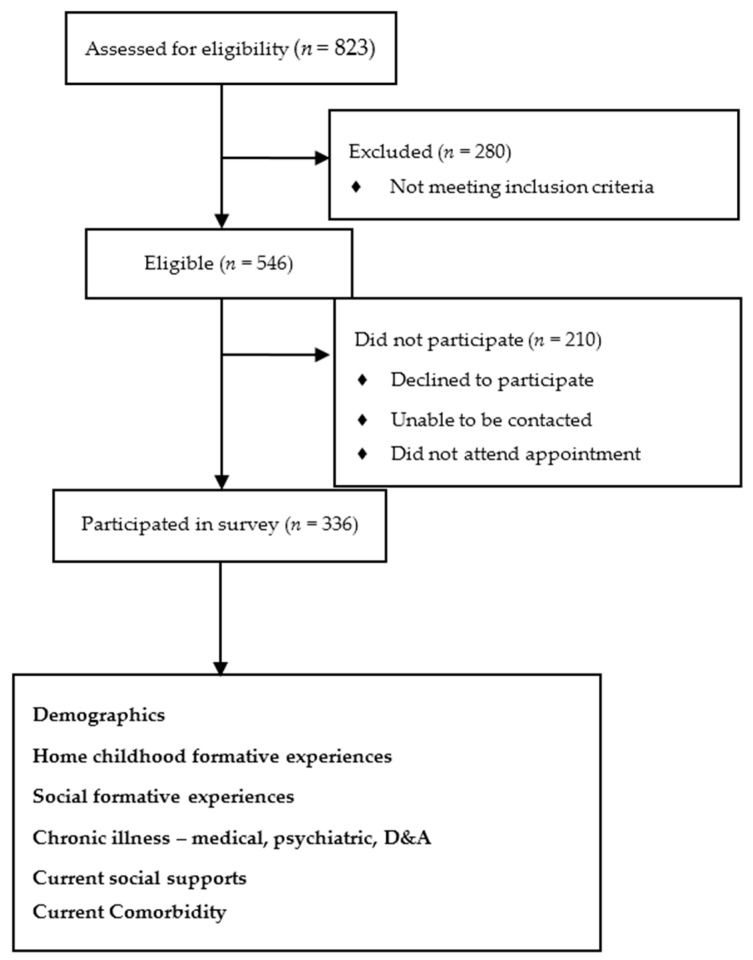
Flow diagram of eligible participants, and enrolment into study.

Research assistants conducted a structured interview with participants, which explored several key domains listed below (See Figure 1).

### 2.1. Outcome Measures

The primary outcome measure for depression utilised the modified Patient Health Questionnaire (mPHQ) as the screening tool for depressive symptoms. This instrument has been validated in an Aboriginal population, with a cut-off score of 9 or more having 80% sensitivity, 71.4% specificity of having a significant depression, with 73.6% concordance with a psychiatric interview [20]. Current suicidal ideation was assessed using item 9 that asked if the participant had thoughts of being better off dead or harming themselves.

The role of childhood trauma was examined with the use of the Childhood Trauma Questionnaire (CTQ), which assesses for positive and negative experiences corresponding to emotional abuse, emotional neglect, physical abuse, physical neglect, and sexual abuse on a 5-point Likert scale, with higher scores corresponding to higher levels of traumatic experiences [21]. It has been validated in various cultural groups, including a younger Aboriginal Australian population [22], and more recently validated in this sample by Radford et al. [15]. The total CTQ score was grouped into tertiles for analysis (lower tertile, mid-tertile, upper tertile).

The AUDIT-C screening tool for high-risk drinking was used to determine high-risk drinking in the past and presently, with a cut-off score of six or more; this is a tool that has been validated in an Aboriginal population [23].

The contribution of resilience was also examined via the use of the Connor–Davidson Resilience Scale (CDRisC), which assesses the degree of resilience based on a self-questionnaire. This has been validated in a sample of older people [24,25], though not in Aboriginal Australians. The total CDRisC score was grouped into tertiles for analysis. Furthermore, experience of racism was a potential factor [26], and was assessed as being having had a previous experience of this, or not. Overall satisfaction with life was assessed utilising the Satisfaction with Life Scale (SWLS), with a cut-off of 26 and above signifying being satisfied with life, and below 26 being dissatisfied with life [27]. This score was then inverted to measure dissatisfaction with life, and how this correlated with current depression.

Cognitive impairment was screened using the Folstein Mini-Mental State Examination [28] with a cut-off score of ≤26 as validated in this same study sample by Radford et al. [29].

### 2.2. Demographic Data

Demographic data included gender, age, location (urban and regional), income (pension and other), marital status (married, separated/divorced/widowed and never married), and accommodation.

### 2.3. Childhood Formative Experiences at Home

Other home formative experiences included parental upbringing (which parent(s) were involved in raising the person), parental loss (if any parent had passed away in childhood), and separation in childhood (separation of the person from family as a child).

### 2.4. Social Formative Experiences

Several relevant measures of adversity that occurred during adolescence and early adulthood were explored. This included school behavioural disturbance (including getting into trouble with school authority resulting in suspension or expulsion and truancy), academic attainment (separated into highest level of schooling, either none, primary, middle, secondary or further education), previous experience of being in custody, and employment history.

### 2.5. Chronic Illness Experiences

Participants were screened using a checklist for the presence of common chronic medical illnesses, which included previous head injury, lung disease, cardiac disease, diabetes, stroke, hypertension, hypercholesterolaemia, cancer, and sleep difficulties (never, infrequent and frequent).

A substance use screen for current, past use and heaviest use including alcohol, smoking, cannabis, and other illicit drugs was employed.

They were also screened for relevant past psychiatric history, including previous depression, anxiety (including stress and PTSD), bipolar disorder and psychotic disorders, and past suicidal behaviours.

“Have you ever been told that you have problems with anxiety, stress disorder or post-traumatic stress disorder/depression/other mood disorders like bi-polar or manic depression/schizophrenia or other similar disorders”?

“Have you intentionally or deliberately hurt or injured yourself (or seriously considered doing this)”?

### 2.6. Psychosocial Factors

Measures of available community support were screened; participants were asked if they felt lonely, and if there were supports they could call upon in the local community.

Their cultural identity and connection were surveyed.

Connected to local Aboriginal community—“Do you feel connected to the local Aboriginal community”?

Strong connection to Country—“How connected to Country to you feel”? 

Traditional language—“Do you speak any Aboriginal Languages”?

### 2.7. Data Analysis

Statistical analyses utilised IBM SPSS v23 (IBM, Armonk, NY, US), with descriptive analyses used for basic demographic data, prevalence of lifetime mental disorders and substance use, and current depression. Odds ratios were calculated in a univariate analysis of current depression using the above predictor variables. A multivariate analysis was also undertaken for current depression, using binomial logistic regression analysis.

In cases with missing values for continuous variables, the median values of the group was utilised where the response rate was at least 75%, before being assessed if they met the threshold for the specific outcome measure. Some participants had carer informants who provided derived data where possible for missing values.

Descriptive analyses were used to display life time prevalence of mental disorders and substance use, as well as the point prevalence of current depression (as utilising the mPHQ described above), current rate of suicidal ideation, cognitive impairment, dissatisfaction with life, and loneliness.

### 2.8. Univariate Analysis

Univariate analysis of current depression was performed using chi-square association with demographic details, childhood experiences at home, social formative experiences (including school behaviour, academic achievement, employment, and custodial experience), lifetime history of mental disorders, substance use and chronic medical conditions, current psychosocial factors, and current comorbidities such as substance use, suicidal ideation and cognitive impairment. Factors with low numbers were excluded, such as history of schizophrenia, bipolar disorder and illicit substances other than marijuana (as marijuana was the predominant illicit substance used). The current use of substances had a high number of non-response, and likewise was excluded from analysis.

### 2.9. Multivariate Analysis

Multivariate analysis was performed with binomial regression analysis utilising factors from the univariate analysis with a cut-off of *p* < 0.1, and co-varied for age and sex. Subsequent regression analysis was performed with factors with a *p*-value < 0.05, until all the remaining factors had *p*-value < 0.05.

## 3. Results

### 3.1. Demographics

There were 823 people initially identified, of whom 280 did not meet inclusion criteria. Of the eligible 546 Aboriginal Australian people, another 210 did not participate for a variety of reasons, including declining to participate, unable to be contacted, did not attend an appointment, or died. Thus, the response rate was *n* = 336 (61.54%). Further details are reported elsewhere in Radford et al. [17].

Demographic details are presented in Table 1.

### 3.2. Prevalence Rates

#### Lifetime and Point Prevalence of Mental Disorders 

The life-time prevalence of mental disorders was anxiety disorders (*n* = 101, 30.1%, (*n* = 13 missing)), depression (*n* = 112, 33.3%, (*n* = 13 missing)), suicidal behaviours (*n* = 64, 19%, (*n* = 14 missing)), bipolar disorder (*n* = 9, 2.7%, (*n* = 16 missing)), and schizophrenia (*n* = 3, 0.9%, (*n* = 16 missing)). The point prevalence of current mental disorders and associated comorbidity is found in Table 2.

### 3.3. Univariate Associations with Current Depression

Factors potentially associated with late life depression were examined from early life childhood formative experiences, social experiences from early, mid, and late life, substance use, and a range of physical and mental health conditions are presented in Table 3. Both risk and protective factors were identified across the age range.

### 3.4. Multivariate Analysis of Current Late-life Depression

Factors for late-life depression from the univariate analysis with *p*-value < 0.1 were further analysed as described in the methodology. The factors that predicted current depression in late-life included having frequent sleep difficulties (OR 3.1, 95% CI 1.5–6.4), previous suicidal behaviour (OR 4.9, 95% CI 2.4–10.3), current suicidal ideation (OR 13.8, 95% CI 5.8–32.9), and living in a regional location (OR 2.1, 95% CI 1.0–4.4) (See Table 4). 

### 3.5. Multivariate Analysis of Suicidal Behaviour History

A similar multivariate analysis was undertaken to examine the factors that predicted having a history of suicidal behaviour. Significant factors included having a history of head injury (OR 2.3, 95% CI 1.2–4.5), having a history of anxiety, stress, and PTSD (OR 5.9, 95% CI 3.1–11.3), and having low resilience on the CDRISC (OR 2.5, 95% CI 1.4–4.8) (See Table 5).

## 4. Discussion

The results from the data demonstrate a high rate of significant depressive symptoms in this NSW sample of older Aboriginal Australian of 18.1%, and suicidal ideations of 11.1%. This cannot be compared directly against other studies due to methodological differences. However, as an approximate comparison to a general cohort of older people, Almeida et al.’s [30] study demonstrated a depression rate of 7.7% in the KICA-Dep, and the ABS [31] had a rate of 2% of suicidal thoughts.

The prominent risk factors associated with late-life depression appears to be living in a regional location, sleep disturbances, life-time history of suicidal behaviour, and current suicidal ideation. Individuals in regional settings are vulnerable to a wide range of inequities of social circumstances, resource allocation, stigmatisation, and accessibility of healthcare [32,33,34], which could be implicated in this finding. Sleep disturbances is often used as a sensitive clinical marker of psychological distress, and has a dose-related relationship with developing and perpetuating late-life depression [35], as well as suicidal ideation [36]; and appears to be a moderate risk factor in this population. Life-time suicidal behaviours and late-life suicidal ideation are strongly predictive of late-life depression, as revealed clinically and in the literature [37,38].

Furthermore, there were several risk factors for having life-time suicidal behaviour, which may in turn be related to developing late-life depression; such as having head injuries, a life-time history of anxiety disorders, and low resilience. This is supported by current literature, with some evidence that past head injuries are correlated to increased suicidal rates [39,40], possibly due to violence that are associated in the environment leading to a head injury, and subsequent psychological distress and levels of hopelessness [41,42]. A recent meta-analysis [43] showed anxiety being a significant predictor of suicidality, particularly for PTSD. Furthermore, low resilience is known to be associated with suicidality, though this may be more significant for mid-life depression [44], and there is mixed evidence that increased resilience may not be protective against suicidal risk [45].

The univariate analysis also demonstrated other factors that were associated with late-life depression, but were no longer statistically significant once adjusted for other factors in the multivariate analysis. However, these findings merit some discussion, as they also have face validity in conferring vulnerability towards late-life depression.

During formative childhood experiences at home, high trauma score on the CTQ was associated with higher late-life depression, particularly for emotional neglect, physical neglect, and sexual abuse. This finding is consistent with studies that demonstrate a clear association between adverse childhood experiences and late-life depression [10,11,46,47]. There was also an association with perceived racism, which has been previously shown by Priest et al. [48] in Aboriginal youths, as being associated with higher rates of anxiety, depression, and suicidal risk, and may be linked with perpetuating a sense of invalidation.

Loneliness in this population was associated with late-life depression, and is consistent with other areas of the literature that demonstrate a graded relationship of loneliness and lack of community support with late-life depression [49], and this could be explained further by recent losses, fewer visitors, and a smaller network [50], and confers a poorer prognosis [51]. Being dissatisfied with life is also associated with depression [52]. Cultural; identity and connectedness did not seem to be statistically significant.

Chronic medical conditions, such as cardiac disease [53] and lung disease [54] are known to be associated with late-life depression. Life-time smoking is weakly associated with late-life depression by virtue of being a cardiovascular risk factor [53]; heavy smoking is known to be associated with elevated psychological distress as well [55].

A factor that appeared protective of late-life depression was death of parent in childhood. A possible explanation may be related to the Aboriginal community that operates in a complex kinship system, which may translate to adult members of the community providing emotional and practical support for bereaved children who have lost their parents, thereby being a buffer against psychological distress. The literature is mixed on this, with some evidence pointing towards later development of depression from death of a parent in childhood, perhaps when associated with other traumatic experiences [56,57], and others that point towards possible source of resilience [58].

**Figure 2:**
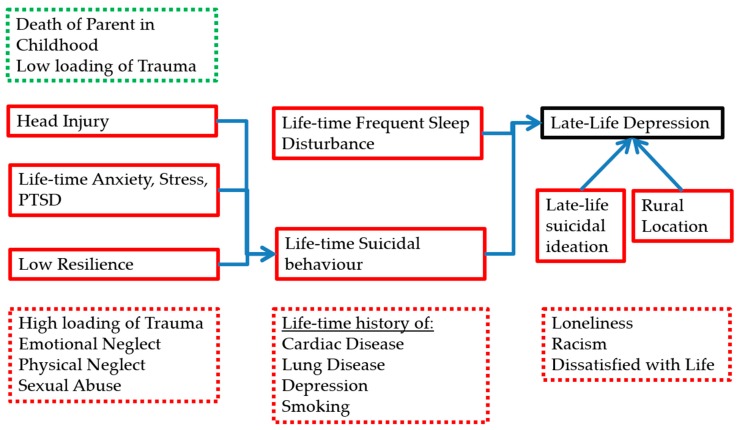
Model of significant risk and protective factors for late-life depression based on the data analyses, whereby associated factors from the univariate analysis are listed in the broken line box and factors from the multivariate analysis are in the solid line boxes. Factors that are associated with depression are in red, and those protective against depression are in green.

A graphical representation of the relevant significant findings can be found in Figure 2.

### 4.1. Limitations

There were certain limitations inherent in this study. There was a lack of a comparison group to compare the significance of these variables.

The structured interview for the collection of historical psychiatric illnesses and childhood factors can be confounded by recall bias, particularly for remote events, particularly in older individuals with varying cognitive difficulties, or recollections which may be associated with shame and embarrassment, lack of mental health literacy, or events which may have been interpreted in a different cultural context, and potential colouring of past life experience if currently depressed; this may lead to inaccurate reporting of the above factors.

The use of the MMSE as a cognitive screening tool may not be the most culturally appropriate cognitive tool to utilize, despite being previously validated in an earlier study [29].

Current depression was defined as a cut-off score based on the mPHQ; while this is a validated screening tool that has a high concordance rate with diagnosis of depression from a psychiatric interview, is not a diagnostic instrument in itself.

The resilience scale (CDRisC) and Satisfaction with Life (SwL) tools have not been validated in an Aboriginal population; while this is not necessarily a draw-back, it is unclear whether there should be any adjustments made for the cut-off scores for being resilient and satisfied, respectively. Other psychosocial factors could have been included, such as cultural identity and activities, community strengths, and access to health resources.

### 4.2. Implications for Prevention and Intervention

This study supports improving the inequities of the regional communities, such as improving resource allocation, recruitment and training of skilled staff, and increased accessibility via novel modes of service delivery such as telepsychiatry, in a culturally appropriate manner, as a strategy to target late-life depression. Furthermore, it should target those at risk of loneliness, racial discrimination, and poor quality of life.

Furthermore, there should be adequate screening and treatment for treatable risk factors for late-life depression, including early onset depression, anxiety, and suicidal behaviours, and chronic medical conditions such as cardiac and lung conditions, and smoking. There should be attention to those with head injuries, childhood trauma, and poor coping factors. There should also be surveillance and increased psychosocial support for vulnerable children at risk from childhood trauma.

## 5. Conclusions

This study’s findings reveal high rates of lifetime prevalence of depression and late-life depression and support the assertion that a life-time history of suicidal behaviours, sleep disorders, regional dwelling, and concurrent late-life suicidal ideation are implicated in late-life depression among older Aboriginal Australians. Current psychosocial factors also play a role in adding to the vulnerability in this population, particularly with loneliness and living in a regional setting. These factors could be potential targets of intervention for prevention of late-life depression in this population, with concerted effort to improve accessibility of screening and treatment to regional communities, increased community supports, and effective interventions for chronic medical, psychiatric illnesses and sleep disorders, and at-risk individuals.

## Figures and Tables

**Table 1:** Demographics.

Variable	*n* (Total *n* = 336)	%	Missing Data (*n*)
Gender			0
Male	136	40.5	
Female	200	59.5	
Age			0
60–69	246	73.2	
70+	90	26.8	
Location			0
Urban	140	41.7	
Regional	196	58.3	
Income source			13
Government Pension	266	79.2	
Other source	57	17	
Marital Status			8
Married	124	36.9	
Widowed, divorced or separated	165	49.1	
Never married	39	11.6	
Accommodation			27
Public Accommodation	175	52.1	
Own Home	81	24.1	
Private Rental	36	10.7	
Residential Aged Care Facility	17	5.1	

Note: The numbers and percentage is based on number of subjects included in the study.

**Table 2:** Point prevalence of current depression and comorbid conditions.

Depression and Comorbidity	*n* (Total *n* = 336)	%	Missing Data (*n*)
Significant Depression on PHQ9	57	18.1	27
Suicidal ideation	35	11.1	21
Cognitive impairment	104	31	
Dissatisfied with life	101	30.1	
Loneliness	162	48.1	23

**Table 3:** Univariate analysis of depression.

Variable (*n*)	Depressed	Not Depressed	Odds Ratio	95% Confidence Interval	Missing Data (*n*)
	*n* (% of variable)				
**Demographics**					
Male (136)	23 (16.9)	113 (83.1)	0.9	0.5–1.5	
Age					
60–69 (245)	44 (18.8)	113 (81.2)	1.2	0.6–2.2	
70+ (91)	15 (16.5)	76 (83.5)			
Regional (196)	44 (22.4)	152 (77.6)	2.1 *	1.1–3.8	
Pension (266)	48 (18)	218 (82)	1	0.5–2.2	13
Marital Status					8
Married (124)	26 (21)	98 (79)	1.3	0.8–2.4	
Widowed, divorced or separated (165)	28 (17)	137 (83)	0.9	0.5–1.5	
Never married (39)	5 (12.8)	34 (87.2)	0.6	0.2–1.7	
Accommodation					27
Private rental (36)	10 (27.8)	26 (72.2)	1.9	0.9–4.1	
Residential facility (17)	2 (11.8)	15 (88.2)	0.6	0.1–1.6	
Public Accommodation (175)	31 (17.7)	144 (82.3)	0.9	0.5–1.6	
Own Home (81)	16 (19.8)	65 (80.2)	1.1	0.6–2.2	
**Childhood Experiences at Home**					
Raised by parents					
Both parents (166)	32 (19.3)	134 (80.7)	1.2	0.7–2	
By mother or father (114)	21 (18.4)	93 (81.6)	1	0.6–1.9	
By neither (56)	8 (14.3)	48 (85.7)	0.7	0.3–1.6	
Death of parent(s) in childhood (74)	6 (8.1)	68 (91.9)	0.3 *	0.1–0.8	2
Taken away from family (33)	8 (24.2)	25 (75.8)	0.6	0.7–3.7	9
CTQ					37
Lower tertile (109)	11 (10.1)	98 (89.9)	0.4 *	0.2–0.8	
Mid tertile (115)	22 (19.1)	93 (80.9)	1.1	0.6–2	
Upper tertile (111)	28 (25.2)	83 (74.8)	2 *	1.1–3.4	
**Social Formative Experiences**					
Behavioural disturbances at school (154)	33 (21.4)	121 (78.6)	1.6	0.9–2.1	11
Highest Education at school					4
Year 6 or less (73)	14 (19.2)	59 (80.8)	1.1	0.6–2.1	
Year 7–9 (190)	34 (17.9)	156 (82.1)	1.1	0.6–2.1	
Year 10–12 (69)	13 (18.8)	56 (81.2)	1	0.5–2	
Further Education (161)	31 (19.3)	130 (80.7)	1.1	0.7–2	1
Previous Employment (302)	57 (18.9)	245 (81.1)	1.3	0.3–5.9	21
Previous Custody (109)	19 (17.4)	90 (82.6)	1	0.5–1.8	13
**Chronic Illness**					
Head injury (95)	19 (20)	76 (80)	1.2	0.7–2.3	10
Stroke (78)	19 (24.4)	59 (75.6)	1.7	0.9–3.1	10
Hypertension (205)	34 (16.6)	171 (83.4)	0.8	0.4–1.4	12
Hypercholesterolaemia (141)	29 (20.6)	112 (79.4)	1.4	0.8–2.4	33
Cardiac Disease (140)	32 (22.9)	108 (77.1)	1.7 *	1–3.1	10
Diabetes (140)	24 (17.1)	116 (82.9)	0.9	0.5–1.6	19
Sleep Difficulties					23
Never (138)	13 (9.4)	125 (90.6)	0.3 *	0.2–0.6	
Infrequent (88)	16 (18.2)	72 (81.8)	1	0.5–1.9	
Frequent (87)	29 (33.3)	58 (66.7)	3.4 *	1.9–6.1	
Cancer (38)	8 (21.1)	30 (78.9)	1.2	0.5–2.8	19
Lung Disease (101)	28 (27.7)	73 (72.3)	2.4 *	1.3–4.3	26
Anxiety, Stress, and PTSD (101)	34 (33.7)	67 (66.3)	4.3 *	2.4–7.9	13
Depression (112)	32 (28.6)	80 (71.4)	2.8 *	1.6–5.1	13
Suicidal behaviour (64)	28 (43.8)	36 (56.3)	5.7 *	3.1–10.6	14
Smoking (228)	47 (20.6)	181 (79.4)	2.1 *	1–4.3	18
Past High-Risk Alcohol Use (187)	31 (16.6)	156 (83.4)	0.8	0.5–1.4	14
Marijuana use (42)	11 (26.2)	31 (73.8)	1.7	0.8–3.6	17
**Current Psychosocial Factors**					
Frequent Loneliness (162)	40 (24.7)	122 (75.3)	3 *	1.6–5.6	23
Community Support (304)	55 (18.1)	249 (81.9)	0.7	0.2–2.5	20
Connected to local Aboriginal community (299)	54 (18.1)	245 (81.9)	0.8	0.3–2.1	9
Strong connection to Country (235)	39 (16.6)	196 (83.4)	0.6	0.3–1.1	29
Traditional language (120)	21 (17.5)	99 (82.5)	1.0	0.5–1.7	13
CDRISC (Resilience)					30
Lower tertile (115)	34 (29.6)	81 (70.4)	3 *	1.2–5.3	
Mid tertile (105)	13 (12.4)	92 (87.6)	0.5	0.3–1	
Upper tertile (116)	14 (12.1)	102 (87.9)	0.5	0.3–1	
Previous Experience of Racism (142)	33 (23.9)	108 (76.1)	2.1 *	1.2–3.8	32
Dissatisfied with Life (209)	28 (27.7)	73 (72.3)	2.3 *	1.3–4.1	
**Current Comorbidity**					
Smoking (87)	17 (19.5)	70 (80.5)	1.1	0.6–2.1	11
High-Risk Alcohol use (31)	8 (25.8)	23 (74.2)	1.7	0.7–3.9	
Cognitive Impairment (104)	19 (18.3)	85 (81.7)	1	0.6–1.8	
Suicidal ideation (35)	22 (65.8)	13 (34.2)	14 *	6.6–29.8	

Results of the univariate analysis. CTQ (Childhood Trauma Questionnaire), PTSD (Post-Traumatic Stress Disorder), CDRISC (Connor-Davidson Resilience Scale). Statistically signfiicant findings are marked with an asterisk (*).

**Table 4:** Multivariate analysis of current depression.

Variable	Odds Ratio	95% Confidence Interval (*p*-Value)
**Demographics**		
Age	0.6	0.03–1.5 (0.29)
Gender	0.9	0.4–1.7 (0.66)
Regional	2.1	1.0–4.4 (<0.05)
Frequent Sleep Disturbance	3.1	1.5–6.4 (<0.005)
Suicidal behaviour History	4.9	2.4–10.3 (<0.005)
Current Suicidal ideation	13.8	5.8–32.9 (<0.005)

**Table 5:** Multivariate analysis of suicidal behaviour history.

Variable	Odds Ratio	95% Confidence Interval (*p*-Value)
**Demographics**		
Age	0.7	0.3–1.5 (0.32)
Gender	0.9	0.5–1.8 (0.76)
Head Injury	2.3	1.2–4.5 (<0.05)
Anxiety, Stress and PTSD History	5.9	3.1–11.3 (<0.005)
Low CDRISC (Resilience)	2.5	1.4–4.8 (<0.005)
